# Incorporation of regulatory DNA elements within a viral vector improves recombinant protein expression in plants

**DOI:** 10.1038/s41598-024-80444-9

**Published:** 2024-11-21

**Authors:** Ryan J. Coates, Simon Scofield, Mark T. Young

**Affiliations:** School of Biosciences, Cardiff University, Sir Martin Evans Building, Museum Avenue, Cardiff, CF10 3AX United Kingdom

**Keywords:** Plant molecular biology, Expression systems

## Abstract

Plants have significant potential as recombinant protein expression chassis, as they can produce complex post-translationally modified proteins that are unobtainable using prokaryotic production systems, with almost limitless scalability and substantially reduced costs relative to eukaryotic cell cultures. Transient protein expression reduces the time taken between transformation and recombinant protein extraction and purification, however low protein yields relative to conventional stable expression systems remain a major obstacle. Here, we have assessed the effectiveness of combining several established genetic components, including a promoter, 5’ UTR, 3’ UTR, double terminator, and matrix attachment region, to modify the TMV-based pJL-TRBO expression vector for improved recombinant protein expression in plants. Using enhanced green fluorescent protein (eGFP) as a reporter, we quantified expression using fluorescence imaging *in planta* together with SDS-PAGE and western blotting and showed that our optimum construct resulted in a significant increase relative to pJL-TRBO-eGFP. This increase was exclusively due to the presence of the additional 5’ UTR. We anticipate that our expression constructs will be a useful tool for high-yield plant recombinant protein production and may serve as a template for further improvements.

**Supplementary Information:**

The online version contains supplementary material available at 10.1038/s41598-024-80444-9.

## Introduction

Recombinant protein production is essential for many medicinal and industrially relevant proteins, with protein drugs accounting for approximately 10% of all drugs in 2017^1^. Traditionally, prokaryotic cell cultures are used to produce recombinant proteins, due to their low maintenance requirements, low costs, and high scalability^2^. However, prokaryotic expression systems cannot produce proteins with complex post-translational modifications (PTMs). Eukaryotic systems can be used to overcome these limitations, but these are typically cell cultures that are expensive to maintain and scale-up. Cytoplasmic proteins can be produced in high quantities in these systems, so typically do not require large volumes of cell culture to produce significant yields. However, proteins with lower intracellular abundance, such as membrane proteins which comprise 26% of the human proteome^3^, require larger volumes of cell cultures to obtain the same final yields of extracted protein. Achieving substantial yields of these proteins often requires scaling up the volumes of these eukaryotic cell cultures, which substantially increases costs. Consequently, some proteins are too costly to produce using conventional expression systems. Fortunately, plants are eukaryotic hosts that can produce complex PTMs, and can be easily scaled up due to their simple and affordable culture requirements. This means that they are potentially ideal production systems for modified membrane proteins and may be a viable alternative for modified cytoplasmic proteins. The cheap upstream scalability of plants, along with the rapid and efficient transformation processes make them ideal candidates for protein production^4^. Furthermore, recent advances in plant protein extraction utilising non-specialist equipment, and the wide availability of soil and water mean that very little expensive or specialist equipment is needed to start producing plant-made recombinant proteins^5,6^.

It has been reported that plant recombinant protein production systems have lower yields per mass unit than conventional systems^7^. However, recent research has led to improvements in these yields, including advances in gene expression, influencing both transcription and translation, reducing protein degradation and gene silencing, and the employment of replicating vectors that improve transient transformation efficacy^5^.

Examples of published improvements include the creation of synthetic DNA elements that improve either transcriptional efficacy, translational efficacy, or transcript stability. Promoters have been designed with expression levels comparable to the commonly used CaMV 35 S promoter^8^. Synthetic untranslated regions (UTRs) have been designed with improved functions over the already powerful CPMV UTRs^9^. Several transcriptional terminators have been identified that improve gene expression, even more so when used in tandem with one another and in combination with matrix attachment regions that increase transcriptional efficiency^10^. Other developments include the use of viral vectors that enable substantially improved expression and transformation efficacies and result in very high levels of protein expression of up to 40% of total soluble protein (TSP) using Tobacco Mosaic Virus (TMV)-derived vectors in some plant species^11^. Deconstructed TMV variants include pJL-TRBO^12^ which lacks the viral coat protein and instead expresses a protein of interest at up to 25% TSP. This vector contains the tobacco mosaic virus replicase proteins and movement protein, enabling amplification of RNA coding for target proteins and subsequent cell-to-cell spread, respectively.

In the present study, we performed a combinatorial analysis of various regulatory DNA elements on the expression of an eGFP reporter gene in transiently transformed *Nicotiana benthamiana* leaves using the replicating viral-derived plasmid backbone pJL-TRBO, which is transmitted from cell-to-cell through plasmodesmata^12^. We have developed expression constructs that demonstrate improved *in planta* and extracted eGFP production compared to the standard pJL-TRBO vector. Our most complex construct combined a promoter, 5’ and 3’ UTRs, double terminator, and matrix attachment region within the pJL-TRBO vector. This produced a ∼ 7-fold increase in eGFP production relative to the pJL-TRBO vector. When investigating the relative effects of the DNA elements, we found that the 5’ UTR was exclusively responsible for this increase. We anticipate that our findings could pave the way for further enhancements in construct design for *in planta* transgene expression, particularly through improving the understanding of the compatibility of different genetic regulatory elements within viral vectors.

## Results

### Incorporation of additional regulatory elements enhances eGFP production in the pJL-TRBO vector

The pJL-TRBO vector^12^ is a deconstructed virus-based vector derived from Tobacco Mosaic Virus (TMV), which improves transformation efficiency through RNA replication and cell-to-cell mRNA movement^13,14^, that has been shown to produce high levels of target protein expression in transiently transformed *Nicotiana benthamiana* plants^12^. The pJL-TRBO vector is normally utilised by inserting the coding sequence of a protein of interest into the multiple cloning site without any additional DNA elements. Other studies have demonstrated that incorporating additional DNA elements into replicating vectors can improve their expression^9,10^, but this has not been tested in the pJL-TRBO system. Consequently, using the pJL-TRBO vector as a backbone, we developed a series of expression cassettes that combine several established DNA elements each shown to improve protein expression, to investigate whetherthese additional regulatory DNA elements would lead to enhanced levels of eGFP expression.

**Fig. 1:**
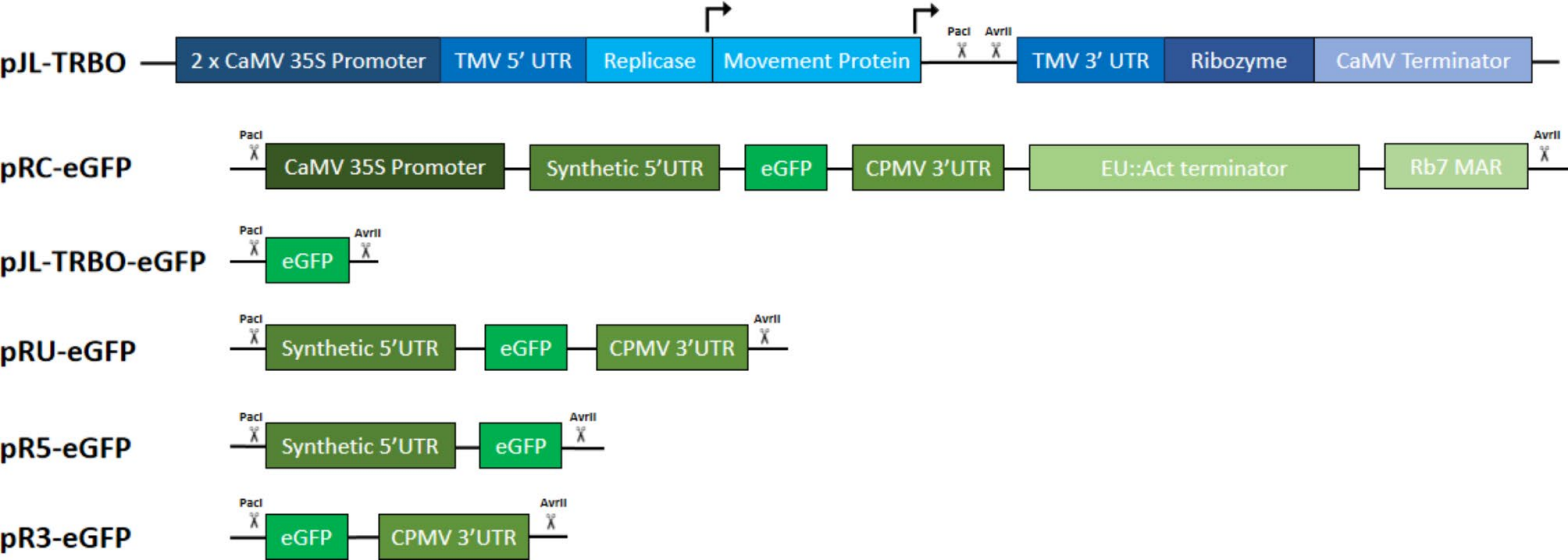
Expression constructs generated and compared in this research. pJL-TRBO = The empty pJL-TRBO vector backbone (blue)^12^ in which the expression constructs (green) were cloned into using PacI and AvrII restriction enzymes (black scissors). pRC-eGFP = Construct containing (5’ to 3’) the CaMV 35 S promoter, a synthetic 5’ UTR^9^, eGFP coding sequence, the CPMV 3’ UTR^9^, *N. benthamiana* Extensin and *N. tabacum* Actin-3 double terminator^10^, and RB7 matrix attachment region^10^ in the replicating vector pJL-TRBO (blue)^12^. pJL-TRBO-eGFP = Isolated eGFP coding sequence in the replicating vector pJL-TRBO. pRU-eGFP = Double UTR construct containing (5’ to 3’) the synthetic 5’ UTR, eGFP coding sequence, and CPMV 3’ UTR in the replicating vector pJL-TRBO. pR5-eGFP = 5’ UTR construct containing (5’ to 3’) the synthetic 5’ UTR and eGFP coding sequence in the replicating vector pJL-TRBO. pR3-eGFP = 3’ UTR construct containing (5’ to 3’) the eGFP coding sequence and CPMV 3’ UTR in the replicating vector pJL-TRBO.

Our most complex construct, pRC, contains an expression cassette that combines the CaMV 35S promoter, a previously published synthetic 5’ UTR derived from the HyperTrans system^9^, the cowpea mosaic virus (CPMV) 3’ UTR^9^, an intronless *N. tabacum* extensin terminator^15^, a *N. benthamiana* Actin terminator^10^, and the RB7 matrix attachment region (MAR)^10^. As the pJL-TRBO vector is derived from an RNA virus, we hypothesised that UTRs would contribute the most to improved gene expression as these are the DNA elements that function primarily at the RNA level through increasing translation and improving transcript stability and accumulation^9^. However, as the native TMV UTRs are essential for the RNA-replication and cell-to-cell movement functions of TMV^13,14,16^, it is possible that the use of additional exogenous UTRs may affect these functions. Thus, to assess whether exogenous UTRs affected eGFP expression, different combinations of the 5’ and 3’ UTRs were also made within the pJL-TRBO vector, generating constructs with both UTRs (pRU), only the 5’ UTR (pR5), or only the 3’ UTR (pR3). All constructs created are shown in Fig. 1 and used an eGFP coding sequence to compare expression.

**Fig. 2:**
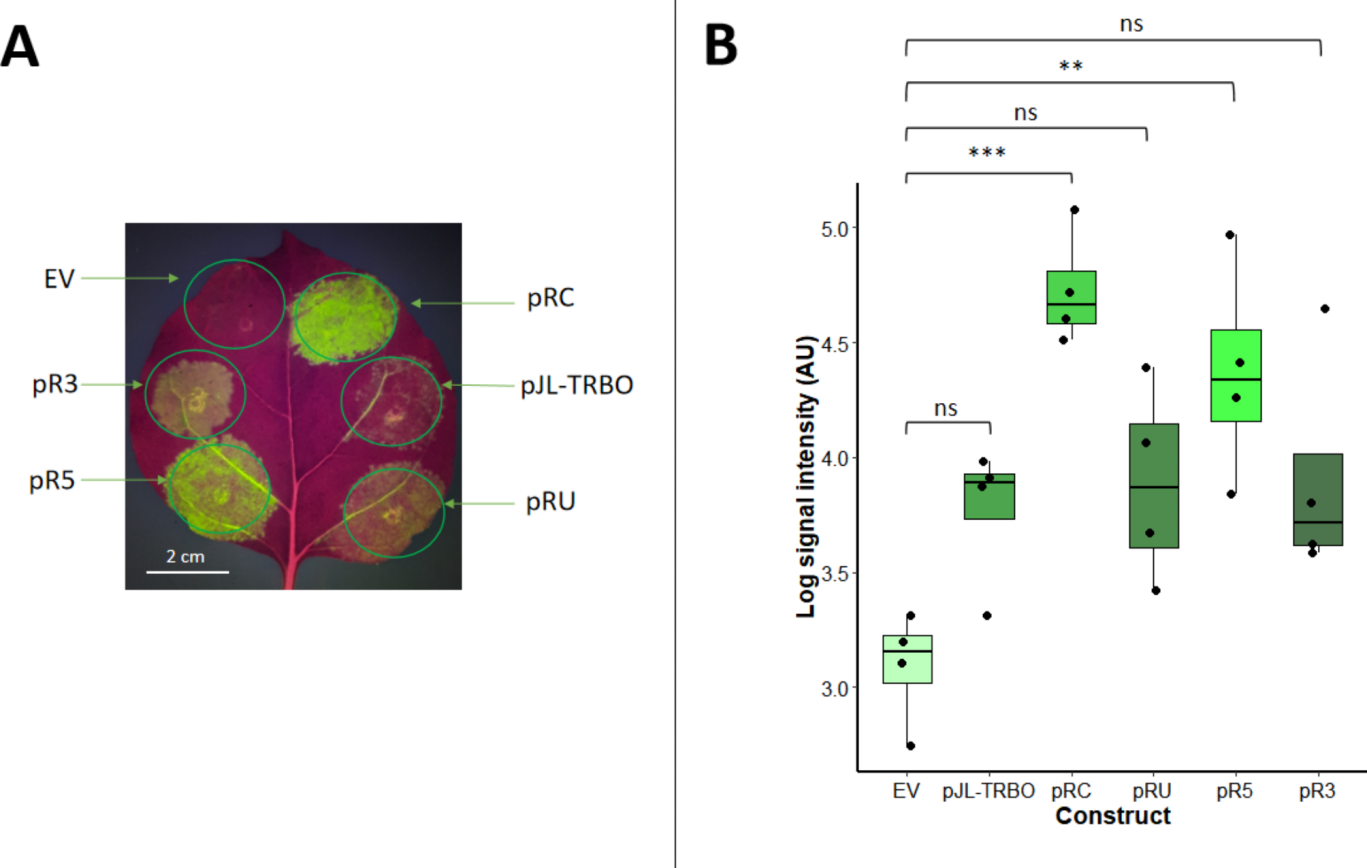
Analyses of all constructs tested simultaneously in single leaves. **A** - representative photograph a single *N. benthamiana* leaf transformed with all eGFP constructs and the empty vector control (EV). **B** - Box plot showing ImageJ quantification of expression from four transformed leaves. A one-way ANOVA showed a significant difference between groups (F_(5, 18)_ = 8.656, *p* = 2.53 × 10^− 4^). Pairwise analyses using a Tukey test showed that only pRC-eGFP and pR5-eGFP produced significantly higher green signal than the empty vector control suggesting that these constructs show the highest fluorescence. The empty vector control showed no visible eGFP fluorescence but a small amount of green signal background is present in all photographs. Each box plot shows the interquartile range for each dataset and the error bars show the standard error of the mean. Individual data points are shown as black dots and the centreline in each plot shows the mean. Significance values are: ns = not significant, * = *p* < 0.05, ** = *p* < 0.01, *** = *p* < 0.001, **** = *p* < 0.0001.

All five of these constructs, plus the empty vector, were transiently transformed into single *N. benthamiana* leaves using *Agrobacterium*-mediated infiltration and visualised 5 days post-infiltration (DPI) under blue light to observe eGFP activity (Fig. 2). Of all the constructs tested, pRC-eGFP and pR5-eGFP produced the highest expression. As a result, these two constructs were carried forward for further testing at larger scales alongside the parent pJL-TRBO-eGFP vector and the empty pJL-TRBO vector control.

**Fig. 3:**
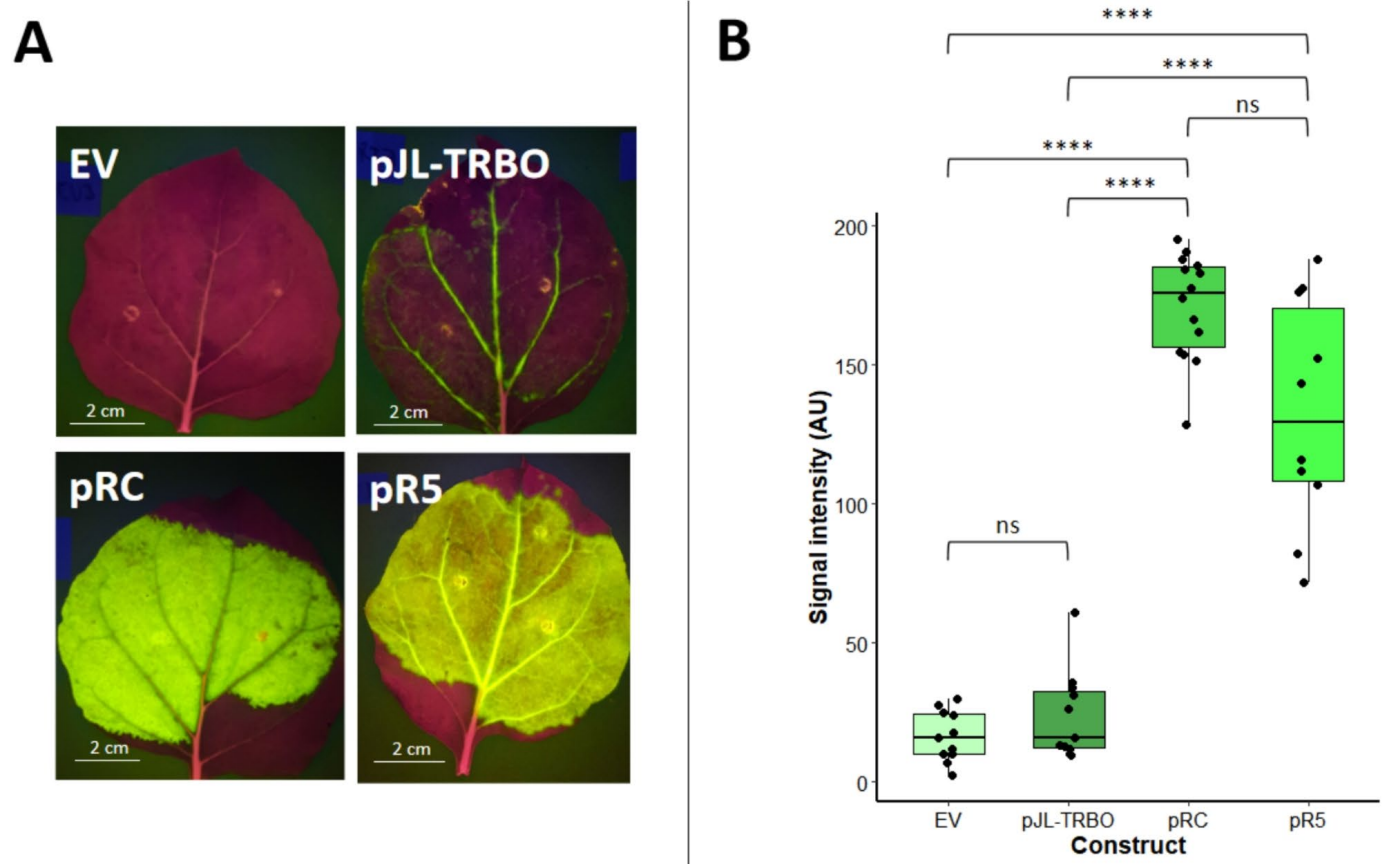
Analyses of the replicating constructs pJL-TRBO-eGFP, pR5-eGFP, and pRC-eGFP with an empty vector control in infiltrated *N. benthamiana* leaves. **A** - Representative photographs showing the eGFP expression from one leaf transformed with each of the constructs under blue light. **B** - Box plot showing ImageJ quantification of the green channel signal intensity from the photographs. The data was not normally distributed with or without data transformations. A Kruskal-Wallis test shows a significant difference between means (H_(3)_ = 35.534, *p* = 9.397 × 10^− 8^). A Wilcox test with bonferroni correction showed that the expression difference between pJL-TRBO-eGFP and the empty vector was not significant (*p* = 1.0). This was true even without bonferroni correction (*p* = 0.27) The expression difference between pRC-eGFP or pR5-eGFP and the pJL-TRBO-eGFP or empty vector leaves was significant (*p* < 0.0001), but the expression difference between pRC-eGFP and pR5-eGFP was not (*p* = 0.11). The empty vector control showed no visible eGFP fluorescence but a small amount of green signal background is present in all photographs. Each box plot shows the interquartile range for each dataset and the error bars show the standard error of the mean. Individual data points are shown as black dots and the centreline in each plot shows the mean. Significance values are: ns = not significant, * = *p* < 0.05, ** = *p* < 0.01, *** = *p* < 0.001, **** = *p* < 0.0001.

When whole leaves from three plants were transformed with each of these constructs, a difference in expression localisation was observed between pRC-eGFP, pR5-eGFP and the parent vector pJL-TRBO-eGFP (Fig. 3A; Fig. S1). Leaves transformed with pRC-eGFP appeared to produce high levels of expression in the leaf lamina, but displayed little if any expression in the vasculature of the leaves. In contrast, eGFP expression induced by pJL-TRBO-eGFP appeared high in vascular tissue, but comparatively low in leaf lamina relative to pRC-eGFP. Strikingly, leaves transformed with pR5-eGFP showed high expression in both the leaf lamina and the vasculature. Quantification of the intensity of green signal (Fig. 3B) showed that pRC-eGFP produced a statistically significant 7.2-fold greater signal than that of pJL-TRBO-eGFP. In addition, pR5-eGFP also produced a significantly higher signal than pJL-TRBO-eGFP (5.6-fold). Although pRC-eGFP gave a 1.29-fold greater signal than pR5-eGFP this difference was not statistically significant. Although eGFP expression is clearly visible in leaves transformed with pJL-TRBO-eGFP, the difference in signal was not significantly higher than the empty pJL-TRBO vector control, likely due to the combination of background green signal in all images, coupled with relatively low expression levels in the leaf lamina. These data demonstrate that the 5’ UTR is the regulatory DNA element that contributes most to the improved expression levels in pRC-eGFP, and perhaps also suggest that the additional DNA regulatory elements in pRC-eGFP may lead to loss of expression in the vascular tissue.

**Fig. 4:**
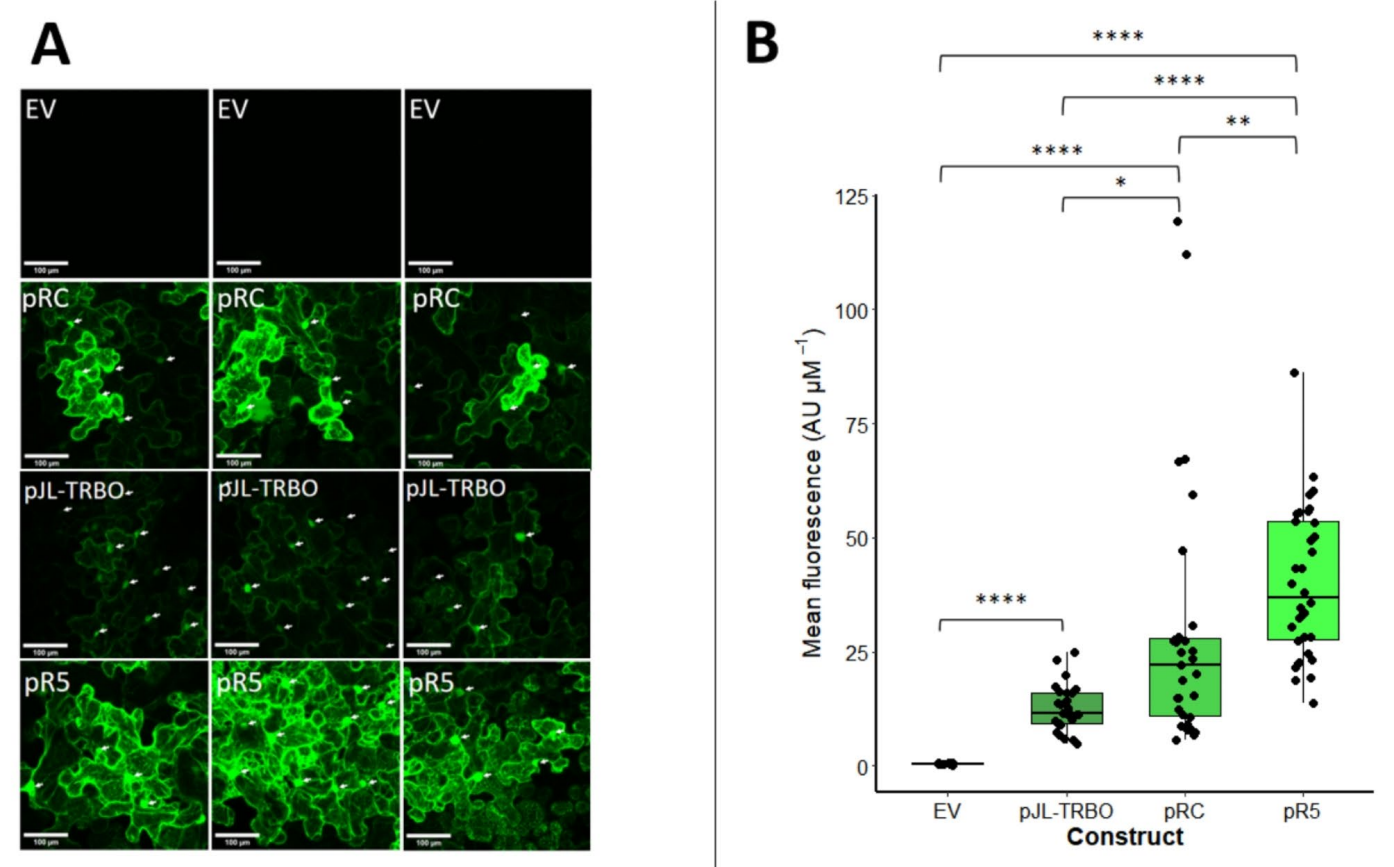
Confocal and per cell expression analyses of the constructs pJL-TRBO-eGFP, pRC-eGFP and pR5-eGFP alongside an empty vector control. **A** – Confocal microscopy images showing 30 μm Z-stacks of transformed areas using a gain of 700 and an excitation wavelength of 488 nm; scale bars represent 100 μm. White arrows indicate nuclei of epidermal cells. **B** - Box plot showing ImageJ per cell quantification of eGFP fluorescence within 10–34 cells transformed with each construct. Data transformations failed to make the data normally distributed or the variances homogenous. A Kruskal-Wallis test shows a significant difference between means (H_(3)_ = 58.965, *p* = 9.782 × 10^− 13^). A Wilcox test with bonferroni correction showed that the expression difference between all constructs and the empty vector was significant (*p* > 0.05). Both pRC-eGFP and pR5-eGFP showed significantly higher fluorescence than pJL-TRBO-eGFP (*p* = 0.0253 and 5.6 × 10^− 13^, respectively). In addition, per cell analyses suggested that pR5-eGFP produced significantly higher fluorescence than pRC-eGFP (*p* = 0.0043). The empty vector control showed no visible eGFP fluorescence. Each box plot shows the interquartile range for each dataset and the error bars show the standard error of the mean. Individual data points are shown as black dots and the centreline in each plot shows the mean. Significance values are: ns = not significant, * = *p* < 0.05, ** = *p* < 0.01, *** = *p* < 0.001, **** = *p* < 0.0001.

The results above suggested that pRC-eGFP and pR5-eGFP produced higher eGFP fluorescence in the leaf lamina than the parent pJL-TRBO-eGFP vector, which primarily resulted in high expression levels in vascular tissue. To quantify eGFP expression at the cellular level in the leaf lamina, laser scanning confocal microscopy was performed on leaf samples transformed with each of the constructs (Fig. 4A). eGFP signal was detected in the epidermal cells of leaves transformed with pJL-TRBO-eGFP, pRC-eGFP and pR5-eGFP but not in control samples transformed with the empty pJL-TRBO vector lacking eGFP. Quantification of eGFP signal in these images showed that all constructs produced significantly higher eGFP fluorescence than the empty vector controls, and both pRC-eGFP and pR5-eGFP produced significantly higher eGFP expression than pJL-TRBO-eGFP. However, the mean expression level for pRC-eGFP was highly variable, and was lower than for pR5-eGFP, which showed more consistent expression levels (Fig. 4B) and showed significantly higher eGFP fluorescence than both pRC-eGFP and pJL-TRBO-eGFP at 1.4-fold, and 3.2-fold, respectively. Together these data suggest that pRC is the most robust construct in driving high levels of target protein expression throughout the leaf tissue, but that pR5 drives the highest per cell expression.

### Expression of eGFP using pRC and pR5 vectors result in improved yields of extracted eGFP protein

**Fig. 5:**
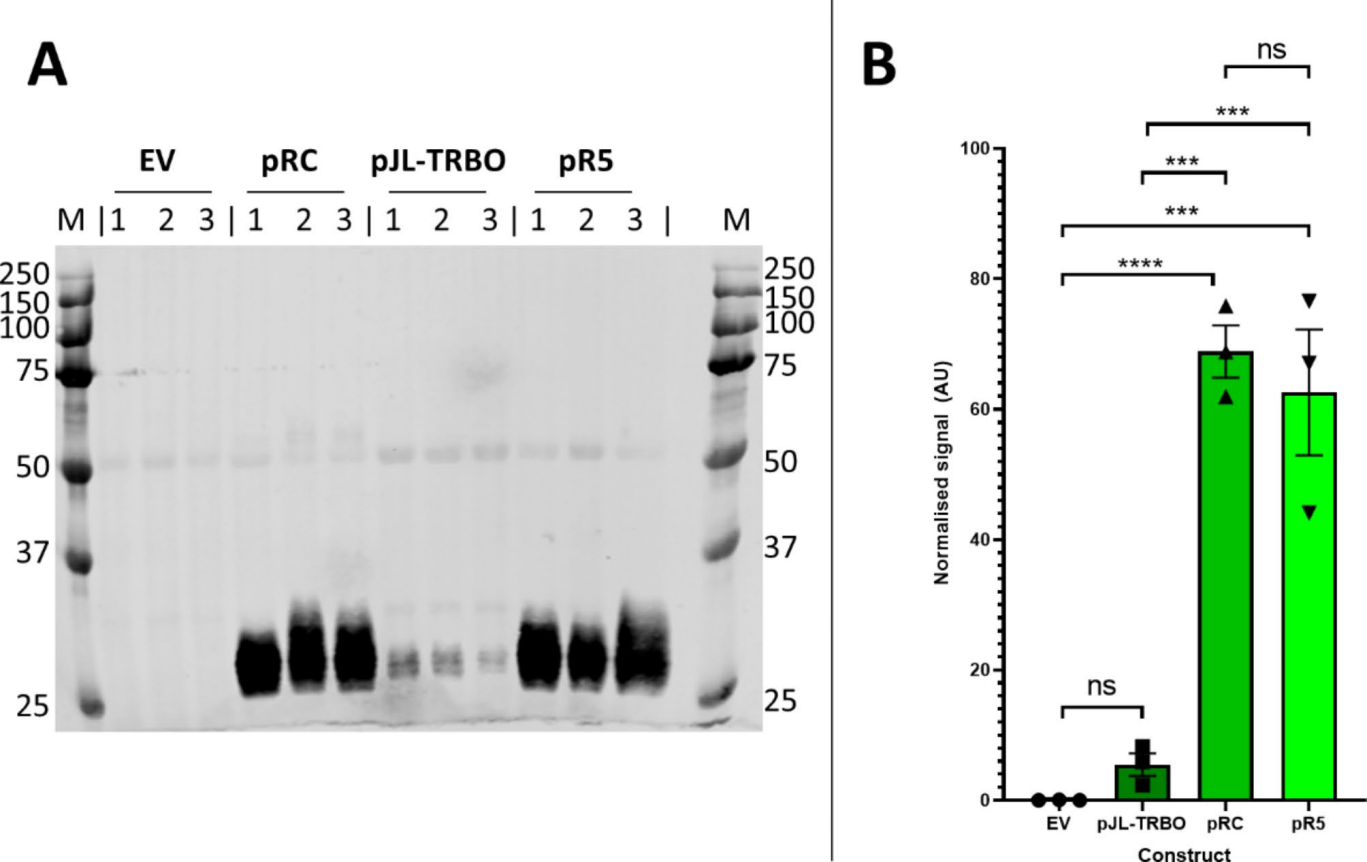
Quantification of extracted protein between constructs. **A** – Raw western blot image showing total protein extracts of plants transformed with empty pJL-TRBO vector, pRC-eGFP, pJL-TRBO-eGFP and pR5-eGFP. Lane M shows a BioRad PrecisionPlus dual colour marker. The eGFP can be seen at 27 kDa. **B** – Histogram showing quantification of the 27 kDa eGFP bands relative to RbcL expression (Figure S3). pRC-eGFP (*p* < 0.0001) and pR5-eGFP (*p* < 0.001) have significantly higher fluorescence than the empty vector control. Both pRC-eGFP and pR5-eGFP show significantly higher fluorescence than the parent pJL-TRBO-eGFP vector (*p* < 0.001). The difference in expression between pRC-eGFP and pR5-eGFP was not statistically significant. Individual data points are shown. Significance values are: ns = not significant, *** = *p* < 0.001, **** = *p* < 0.0001.

Finally, experiments were performed to compare if yields were similarly improved when eGFP protein was extracted. Total protein was extracted from transformed plants and analysed by SDS-PAGE followed by a western blot with a plant-specific eGFP antibody or InstantBlue™ staining for total protein (Fig. S3). Comparison of constructs by Western blot (Fig. 5A) showed similar levels of expression in pRC-eGFP and pR5-eGFP, but both showed much greater signal intensity than pJL-TRBO-eGFP. There was no detectable signal in the empty vector control. Quantification of this signal relative to the large subunit of RuBisCO (RbcL) to control for protein loading (Fig. 5B, Fig. S3) showed that both pRC-eGFP and pR5-eGFP produced significantly higher eGFP expression than pJL-TRBO-eGFP at 14- and 12-fold, respectively. The difference in expression between pRC-eGFP and pR5-eGFP was not significant. Protein extracts from leaves transformed with pJL-TRBO-eGFP produced significantly higher fluorescence than those from leaves transformed with the empty vector control. Quantification of the expressed eGFP relative to RbcL after subtraction of the empty vector control lanes from band densities obtained for eGFP expression (Figure S3C) gave eGFP: RbcL ratios for pJL-TRBO, pRC and pR5 of 0.16, 0.32 and 0.36 respectively. Assuming that RuBisCO is reportedly expressed at approximately 30% of total soluble protein (TSP) in Nicotiana plants^17–19^, then RbcL would represent 24.6% TSP (3.7 μg protein per lane (we loaded 15 μg). From this, we estimate that, per lane, there is 1.18, 0.60 and 1.33 ug eGFP for pRC, pJL-TRBO and pR5 respectively. Therefore, we can report expression of 79 ug per mg soluble protein for pRC (∼ 8% TSP), but it is important to note that for accurate quantification we would need to purify the expressed eGFP and measure it on the same SDS-PAGE as known eGFP standards. Together these data suggest that the additional DNA elements in both pRC and pR5 results in improved target protein expression with the pJL-TRBO vector, but that the 5’ UTR is likely exclusively responsible for this.

## Discussion

In this work we have demonstrated that incorporating additional DNA elements within the TMV-based replicating vector, pJL-TRBO, improves target gene expression both *in planta* and at the extracted protein level. We tested several DNA elements in a complex construct, pRC which utilised several additional DNA elements including a promoter, two terminators, 5’ and 3’ UTRs and a matrix attachment region. This construct improved gene expression by significantly relative to the parent vector, pJL-TRBO.

We hypothesised that UTRs were the DNA element most likely able to improve the expression within replicating vectors. In particular, the synthetic 5’ UTR is derived from the already powerful HyperTrans system, which drives very high levels of gene expression in plants^9,20^. To test this, we developed a series of constructs that utilised these different UTRs and found that the 5’ UTR did indeed lead to significantly increased eGFP expression, as pR5-eGFP increased extracted eGFP by 14-fold compared to the pJL-TRBO-eGFP vector which lacked the UTR. Importantly, the constructs containing either the CPMV 3’ UTR (pR3-eGFP) or both UTRs (pRU-eGFP) had reduced expression relative to pR5-eGFP, suggesting that the 3’ UTR is not responsible for the expression increase. In future work, correlation of eGFP mRNA levels with eGFP fluorescence or extracted eGFP protein levels will help us to understand the potential impact these UTR sequences have on mRNA stability or translation efficiency.

Interestingly, the construct containing all the regulatory elements, pRC-eGFP, did not display significantly different eGFP expression levels to the pR5-eGFP construct, suggesting that the additional DNA elements are not detrimental to the expression. However, the use of these additional DNA elements appeared to change the expression pattern within the leaf. Transformation using the parent pJL-TRBO-eGFP vector resulted in high expression primarily in the leaf vasculature, though we were still able to detect expression in the leaf lamina using confocal microscopy. In contrast, using pRC-eGFP resulted in high expression levels in the leaf lamina but little expression in the vasculature. Leaves transformed with pR5-eGFP had high levels of expression in both the leaf lamina and the vasculature. Vascular expression can also be seen in leaf sections transformed with pRU-eGFP and pR3-eGFP, suggesting that the 3’ UTR is not responsible for the localisation difference in pRC-eGFP. This suggests that the additional promoter, terminators or MAR within pRC affected the expression pattern and/or transformation efficiency or viral movement in a tissue-specific manner. This is unusual, as the additional CaMV 35 S promoter is thought to be a constitutive global promoter that drives expression in most tissues and is used to drive constitutive global expression within the parent pJL-TRBO construct^12,21^. Consequently, it is likely that the presence of the terminators or MAR causes this difference in expression localisation. Supporting this, a similar expression pattern can be seen in transformed leaves in the work by Diamos and Mason^10^, where these terminators and MAR are used, however the reason for this expression pattern was not discussed. It is unlikely that the MAR influences the expression pattern as it is thought to buffer the transgene from the surrounding chromatin environment within the nucleus to drive transgene expression regardless of the cell type and surrounding chromatin state^22^. The constructs used by Diamos and Mason^10^ which lacked MARs also had this expression pattern suggesting that the terminators may be the cause. In support of this, it has been reported that terminator activity can affect transcript accumulation in different tissues^23^ which can be mediated by regulation of transgene silencing^24^. Thus, it is likely that the additional terminators within pRC-eGFP cause this tissue-specific change in expression, although the mechanism by which this occurs is unknown.

Other studies have demonstrated that replacing the CPMV 3’ UTR with the TMV 3’ UTR abolished expression within CPMV, showing that native UTRs can be essential for viral vector function^25^. Conversely, in this research the TMV UTRs were not removed but instead combined with an exogenous CPMV-derived 5’ UTR into the TMV-based expression vector (pR5-eGFP) which significantly improved expression relative to parent pJL-TRBO-eGFP vector without any exogenous UTRs. This suggests that chimeric replicating vectors could result in improved expression depending on the parts used and their orientation, shedding light on the complexity of combining different DNA elements within replicating vectors and showing that certain combinations can have unexpected effects.

We anticipate that future improvements to our expression constructs will be possible, particularly with the use of synthetic DNA elements with enhanced functions, which have had moderate success in recent years and are likely to see further improvements. These include using synthetic promoters^26,27^ and promoter stacking^28^, alternative terminator pairs^10^ and the use of introns, known to improve expression through intron mediated enhancement^29^. Additionally, it is well established that co-expression of viral silencing suppressors can improve transgene expression, up to 3-fold in some research^30^. As a result, it is likely that optimisation of silencing suppressor co-expression could further improve the expression levels seen here.

It is important to note that the generated constructs have thus far only been tested using eGFP as the expressed protein, and the construct efficacies may be affected by the nature of the protein that is expressed. Furthermore, if a protein requires post-translational modifications, then the protein processing may incur a significant production bottle-neck. As such, the compatibility of the system with alternative proteins should be tested in the future.

## Materials and methods

### Gene synthesis

Synthesis of DNA was performed externally by GeneWiz^®^. All genetic sequences can be found in the supplementary material. The eGFP sequence (https://www.fpbase.org/protein/egfp/) is derived from GFP (UniProt Accession: P42212). DNA sequences have been deposited to Genbank using Bankit submission ID 2852148. Genbank accession numbers are as follows: 35S CaMV Promoter = PQ062121, 5’ UTR = PQ062122, 3’ UTR = PQ062123, Actin Terminator = PQ062124, Extensin terminator = PQ062125, MAR = PQ062126.

### PCR amplification

PCR amplification was carried out using Q5 DNA polymerase according to manufacturer’s instructions. Primer sequences used can be found in the supplementary material.

### Cloning

Golden Gate cloning^31^ was achieved by mixing 1.5 μL of T4 DNA ligase buffer, 1 μL Type IIS restriction endonuclease (either BsaI or BpiI), 1 μL of T4 DNA ligase, vector: insert in a 1:3 molecular ratio, and ddH_2_O to a total volume of 15 μL. These were placed in a thermocycler for 10 cycles of 37°C for 5 minutes, then 16°C for 5 minutes, followed by a final 37°C cutting step for 10 minutes, then a 55°C followed 80°C denaturation step, each for 10 minutes, with a final hold of 4°C. Conventional restriction endonuclease cloning was carried out by mixing 1 x NEB rCutSmart™ Buffer with 0.5 μL of appropriate restriction enzyme, 500 ng of DNA and ddH_2_O to a total volume of 10 μL and incubated at the optimal temperature of the restriction endonuclease for 1 hour. The digest reaction was separated on an agarose gel and the appropriate band extracted from the gel. Ligation reactions were carried out using an approximate 3:1 Insert: vector (pICH47732) ratio at maximum concentration, 1 x T4 DNA ligase buffer, 0.5 μL DNA ligase and ddH_2_O to a final volume of 10 μL and left overnight at 16°C. Expression cassettes were then PCR amplified from the purified cloning vector using overhands that add PacI and AvrII restriction sites to the 5’ and 3’ ends, respectively. These were then digested and ligated into the pJL-TRBO vector as described above. pICH47732 was a gift from Sylvestre Marillonnet (Addgene plasmid # 48000 ; http://n2t.net/addgene:48000; RRID: Addgene_48000), and pJL-TRBO was a gift from John Lindbo (Addgene plasmid # 80082 ; http://n2t.net/addgene:80082; RRID: Addgene_80082).

### Transformation of bacteria

Mix ‘n’ go *E. coli* (DH5α) were transformed using manufacturer’s instructions (Zymoresearch, catalogue number: T3007). 50 μL aliquots of *Agrobacterium tumefaciens* (Str. GV3101; VWR, catalogue number: 103753-234) were thawed on ice for approximately 10 min. 150ng of DNA to transform was added to each aliquot. The solution was gently pipetted into a BIO-RAD Gene Pulser^®^ Cuvette (catalogue number: 165–2086), and the cells were electroporated at 2500 volts until completed, indicated by the BIO-RAD MicroPulser™ machine. 1 mL of LB was added, and the solution was transferred to an Eppendorf tube, and incubated at 28 °C for 2 h. Cells were then plated onto agar plates containing appropriate antibiotic.

### Growth of bacteria

*E. coli* were grown at 37 °C for 16–24 h, and *A. tumefaciens* were grown at 28 °C for 40–48 h in LB media or on LB agar plates containing appropriate antibiotic.

### Extraction of DNA

Plasmid DNA was extracted from bacteria using a QIAprep Spin Miniprep Kit (Qiagen, catalogue number: 27106), and from agarose gels using a Zymoclean Gel DNA recovery kit (Zymoresearch, catalogue number: D4001/D4002), both according to manufacturer’s instructions.

### Growth of *N. benthamiana*

Plants were grown at 25 °C in soil and sand in a 3:1 ratio. The soil was heat treated prior to potting at 70 °C for at least 2 h. All plant research was conducted in compliance with international and UK guidelines. No endangered species were used in this research.

### Transformation of *N. benthamiana*

Six-week-old *N. benthamiana plants* were used for transient transformation. *Agrobacterium* containing the expression construct of interest were grown for 48 h in 50 mL LB containing appropriate antibiotic in a sterile volumetric flask with foil on top allowing aeration. Cells were transferred to a 50 mL falcon tube and pelleted by centrifugation at 10,000 x g, at 4 °C for 20 min. The supernatant was discarded, and the cells were then diluted in activation buffer (10mM MgCl_2_, 10 mM MES, 200 μM acetosyringone, pH 5.6) to an OD600 of 1.0. The activated *Agrobacterium* were left at room temperature for two hours. For screening experiments, four leaves from different plants were syringe-infiltrated with all of the constructs and empty vector control in small circles across the leaf. When whole leaves were transformed with single constructs 10–14 leaves from 3 different *N. benthamiana* plants were syringe infiltrated with each of the constructs maximising the transformed area in each leaf. Transformed leaves were analysed at 5 days post-infiltration (DPI).

### Photography and analysis of leaves under blue light

Transformed leaves were analysed 5 DPI under blue light using a Dark Reader™ (Clare Chemical Research, Catalogue number: DR89X). Leaves were viewed using an orange filter to visualise eGFP expression and photographed using a Samsung galaxy A51 with an International Organization for Standardization (ISO) sensitivity of 640, a 1 s exposure time, and a stand set approximately 40 cm away from the leaf. Photographs were imported into ImageJ where the red, green and blue channels were split into grey-scale images. A representative image of each channel can be seen in Figure S2. Using the green channel, transformed regions were manually selected and the mean signal intensity was quantified using the ImageJ measurement tool.

### Confocal microscopy

A Zeiss LSM 880 confocal microscope with Airyscan was used. A 1 cm^2^ section of interest was excised and placed on a glass slide with a droplet of water followed by a cover slip and visualised. Confocal images were taken using Z-stacks of 30 μm using a pinhole size of 0.8 Airy Units to generate a 1.7 μm section on a 20-times magnification. Laser excitation of eGFP was achieved using a wavelength of 488 nm with a gain of 700. Dimensions for images were X = 1024, Y = 1024. Images were processed using FIJI and maximum intensity projections of Z-stacks are shown for qualitative images. For quantitative images, Z-stacks were not combined, and instead individual images were scanned to identify the centre of the nucleus, the brightest region within transformed cells. Regions of interest (ROIs) were manually selected following the cellular perimeter and measurements were obtained to gather the area, mean expression, and integrated density. This was performed for both the nuclei of cells and the surrounding cytoplasm within only the central plane in 2D. 3D images were not obtained which would allow quantification of cellular fluorescence across all planes. Full microscopy settings for using the Zeiss LSM 880 confocal microscope can be found in the supplementary material.

### Protein extraction

Eight-week-old plant tissue, one week after transformation, was finely ground in liquid nitrogen using a pestle and mortar. The resulting powder was resuspended in 4 °C lysis buffer (50 mM Tris-HCl, pH 7.5, 165 mM NaCl, 5 mM DTT and 1 x Sigma Plant Protease Inhibitor Cocktail (Sigma Aldrich, Catalogue number: P9599)), in a 1:4 w/v ratio. The tissue was then filtered through double-layer Miracloth (Sigma Aldrich, Catalogue number: 475855-1R) to remove heavy debris. The resulting filtrate was then centrifuged at 4,000 x g for 15 min in an ultracentrifuge to remove debris, and the supernatant containing the crude protein extract transferred to a fresh centrifuge tube.

### Quantification of plant protein extracts

The protein concentrations of plant fractions were analysed using a Bio-Rad protein assay. Briefly, 15 μL of sample (either pure or diluted 1:10, sample: ddH_2_O) or BSA standard (500, 250, 125, 67.5, and 31.25 μg/ml) were gently mixed with 200 μL of 1 x Bio-Rad Protein Assay solution (Bio-Rad, Catalogue number: 5000006) in a transparent 96-well plate. The plate then had absorbance read using a Clariostar Plate Reader. A BSA standard curve was generated, and the sample protein concentrations plotted against it. In addition, the samples were also analysed for GFP-fluorescence in the Clariostar Plate Reader, using a gain of 1250. Statistical analyses were carried out to identify whether the difference in fluorescence was significant to the BSA standards.

### SDS-PAGE

SDS-PAGE was performed using 12% hand-cast polyacrylamide gels (resolving gel = 1.7 mL dH_2_O, 1.3 mL 1.5 M Tris-HCl, pH 8.8, 2 mL 30% Acrylamide (37.5:1), 25 μL 20% SDS, 17 μL 30% ammonium persulfate, 4 μL TEMED. Stacking gel = 1.4 mL dH_2_O, 250 μL 0.5 M Tris-HCl, pH 6.8, 330μL 30% acrylamide (37.5:1), 10 μL 20% SDS, 7 μL 30% ammonium persulfate, 2 μL TEMED). 10 μg of denatured protein sample was loaded into each well as quantified by Bio-Rad protein assay. 3 μL of Precision plus dual color marker (Bio-Rad) was loaded into one or two wells on each gel. Samples were separated using a voltage of 90 V for approximately 2.5 h. Gels were stained for total protein using InstantBlue (Abcam).

### Western blotting

The SDS-PAGE gel was carefully transferred to a nitrocellulose membrane and placed into a Trans-Blot^®^ Turbo™ Transfer System (BIO-RAD, catalog number 1704150) on the 7 min turbo setting to transfer the proteins to the membrane. The membrane was then blocked in Intercept^®^ (TBS) Blocking Buffer (LI-COR, catalog number 927-60001) by rotating in 10mL of the solution at room temperature for 1 h. Following this, the membrane was left in primary antibody solution (10 mL Blocking buffer containing 2% Tween (v/v) and primary antibody (Anti-GFP (Plant Specific) Antibody; antibodies.com, catalog number A50024) at a concentration 1/5000 v/v) overnight at 4 °C. The next day, the membrane was washed 4 times in 10 mL TBS containing 2% v/v Tween, each for 5 min rocking at room temperature. Following, the secondary antibody solution was added (10 mL Blocking buffer containing 2% Tween (v/v) and secondary antibody (Goat anti-Mouse IgG (H + L) Highly Cross-Adsorbed Secondary Antibody, Alexa Fluor™ Plus 800; Invitrogen, catalog number A32730) at a concentration of 1/13350 v/v), and incubated, rocking, for an hour at room temperature. The membrane was then washed four times again in TBS-tween solution and visualised using an Odyssey^®^ CLx Imaging System and converted to grey-scale. .

### Statistical analyses

Statistical analyses were performed using R version 4.4.1 (2024-06-14 ucrt) -- “Race for Your Life”. All data were analysed for a normal distribution using a Shapiro Wilks test and for homogeneity of variances using a Bartlett’s test. For the data in Fig. 2, Panel B, a logarithmic data transformation was applied as this made the data normally distributed according to a Shapiro-Wilks test (W = 0.98065, *p* = 0.9076) and the variances homogenous according to a Bartlett’s test (X^2^_(5)_ = 2.5474, *p* = 0.7963) enabling the use of parametric statistical tests. The data shown in Figure S3, Panel D also had a logarithmic data transformation applied as this made the data variances more homogenous and suitable for parametric analyses according to a Bartlett’s test (X^2^_(3)_ = 6.6235, *p* = 0.08492). For the data seen in Figs. 3 and 4 no data transformations were applied and non-parametric statistical tests were used instead.

## Electronic supplementary material

Below is the link to the electronic supplementary material.

Supplementary Material 1

## Data Availability

The raw data supporting the conclusion of this article will be made available by the authors upon request. Please contact the corresponding author (ScofieldS@cardiff.ac.uk). The DNA sequences generated and/or analysed during the current study are available via Genbank (https://www.ncbi.nlm.nih.gov/genbank/) using Bankit submission ID 2852148. Genbank accession numbers are as follows: 35S CaMV Promoter = PQ062121, 5’ UTR = PQ062122, 3’ UTR = PQ062123, Actin Terminator = PQ062124, Extensin terminator =PQ062125, MAR = PQ062126.
