# Supplementary material for: Incorporation of regulatory DNA elements within a viral vector improves recombinant protein expression in plants
Source: Sci Rep. 2024 Nov 21;14:28865. doi: 10.1038/s41598-024-80444-9 (PMC11582814; doi:10.1038/s41598-024-80444-9)
Supplement: Supplementary file 1 — Supplementary Material 1 [file 41598_2024_80444_MOESM1_ESM.docx]

**Supplementary Information**


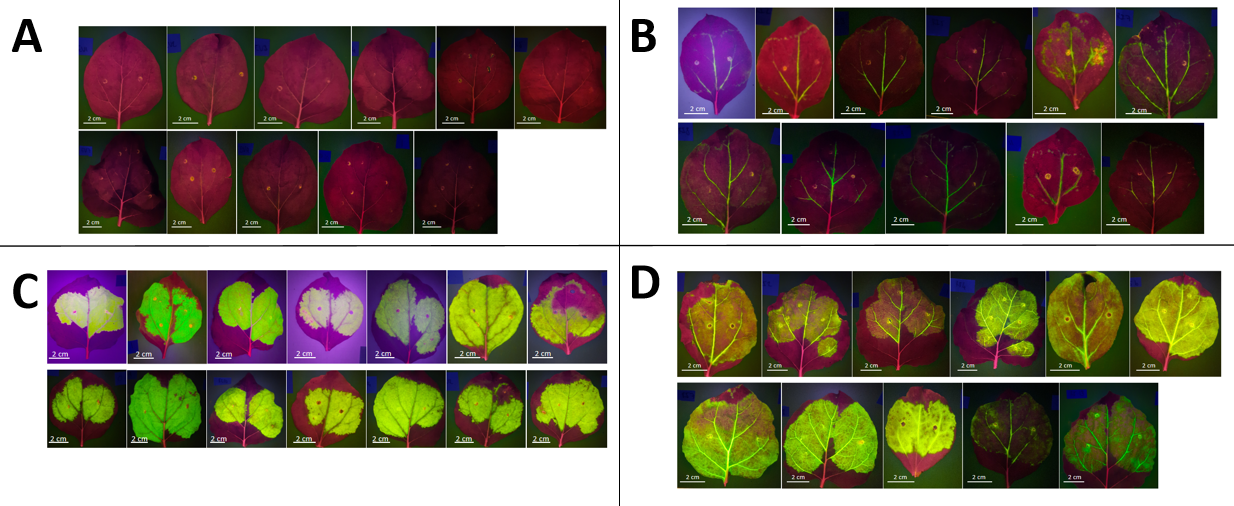


**Figure S1: Images showing the eGFP expression from all leaves transformed with each of the constructs under blue light.** 10-14 leaves from 3 different plants were syringe infiltrated with each of the constructs maximising the transformed area in each leaf and analysed 5 DPI under blue light. A - Empty pJL-TRBO vector. B – pJL-TRBO-eGFP. C – pRC-eGFP. D- pR5-eGFP.


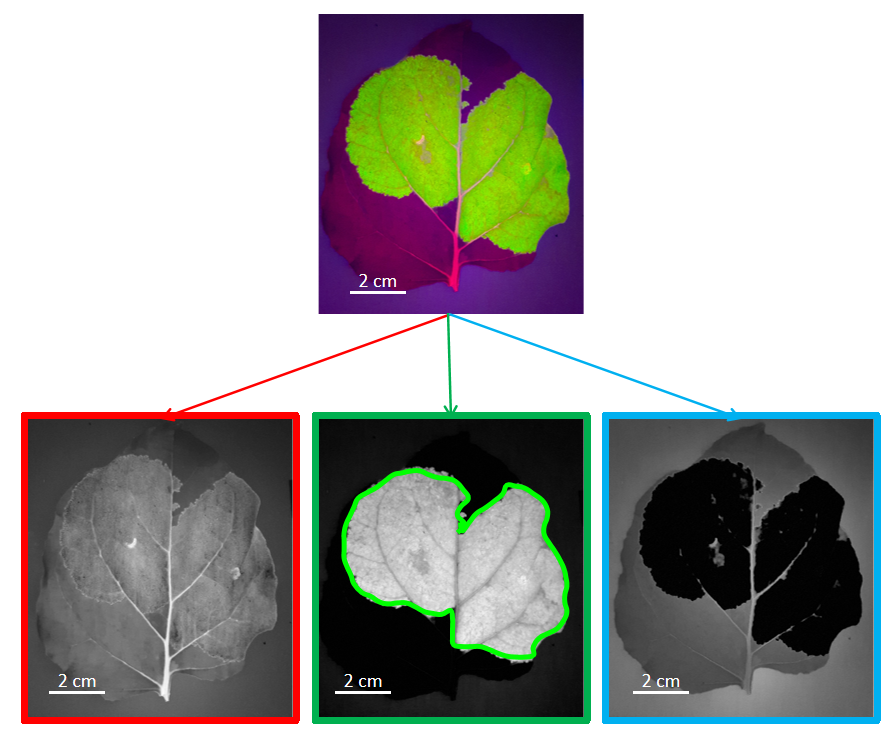


**Figure S2: Representative image of one leaf split into its three colour channels.** Each leaf photograph had green signal measured in this way. Coloured photographs were split using ImageJ into their three red (left), green (centre) or blue (right) colour channels. To measure the eGFP expression, the transformed area of the green channel was highlighted and measured, shown by the bright green outline.


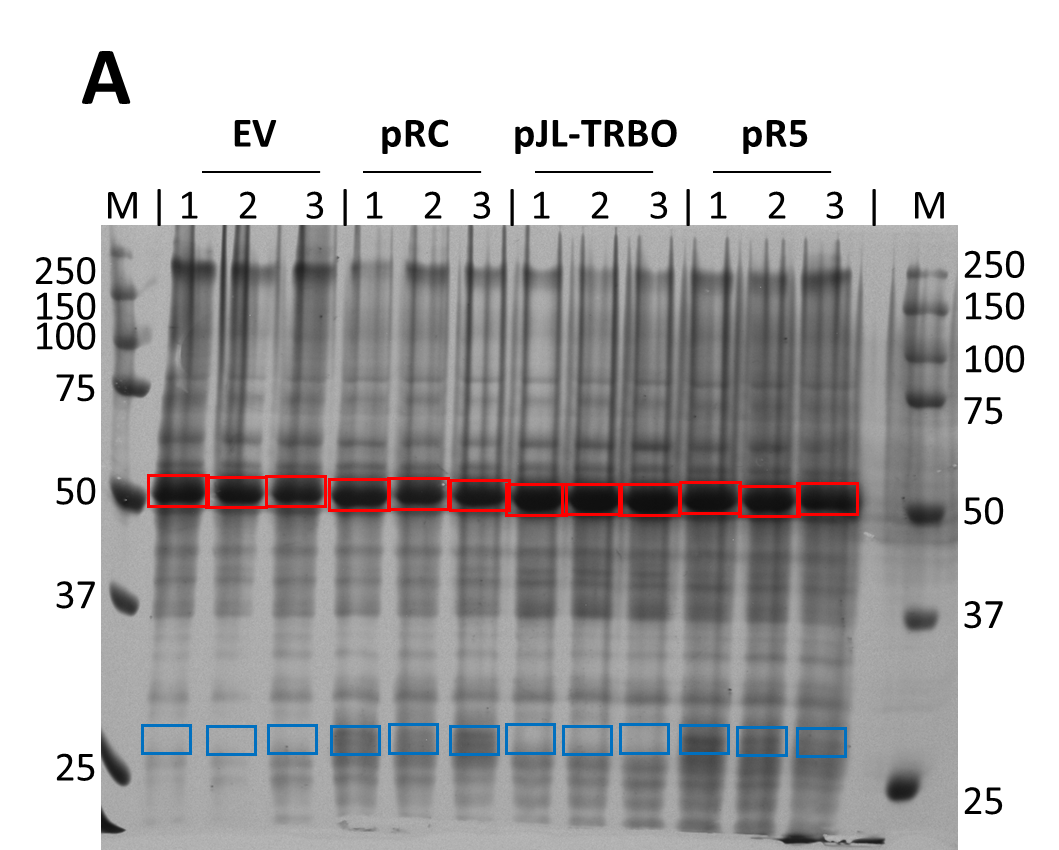

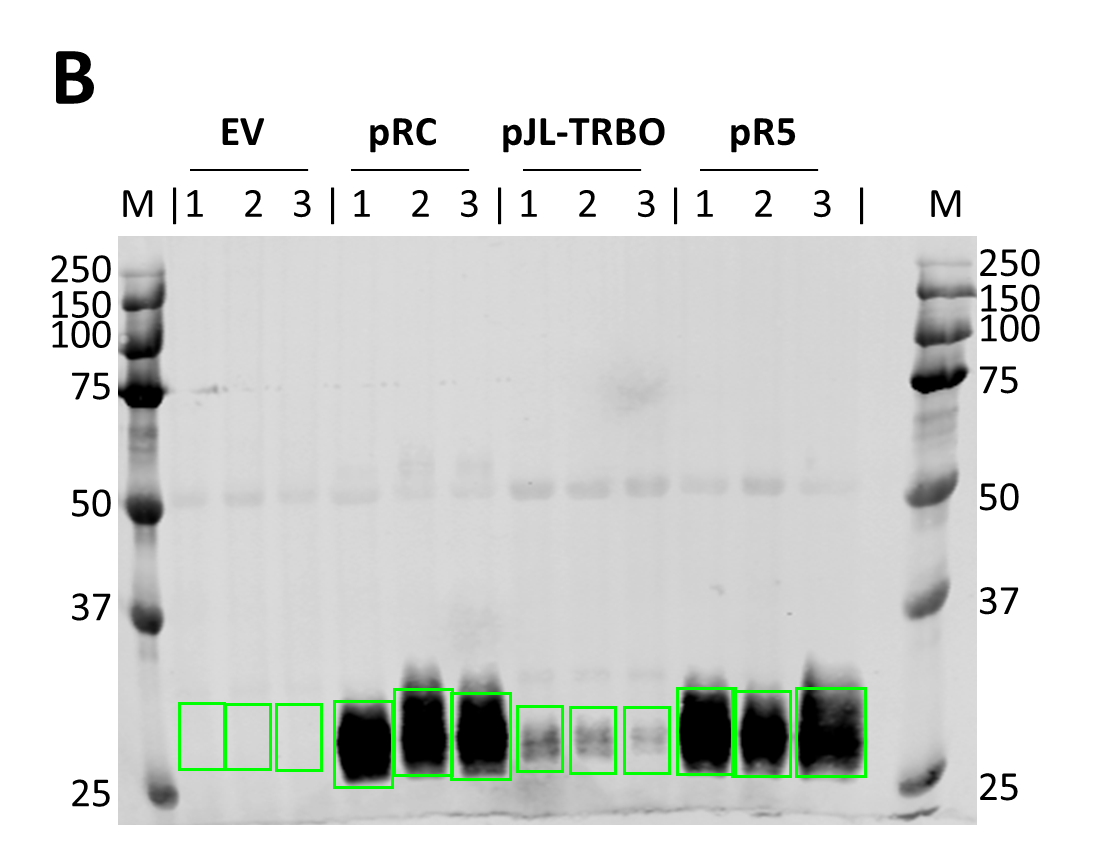


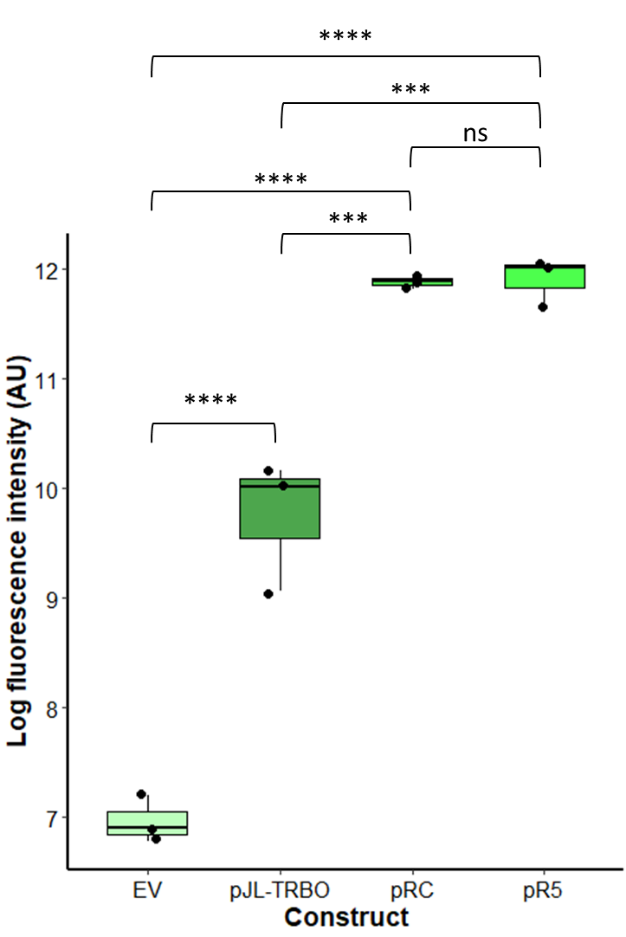


**D**

**Figure S3. Original InstantBlue and Western Blot images (uncropped) used for data quantification and for Figure 5**. **A.** InstantBlue-stained gel (15µg protein loaded per lane) showing total protein expression (in triplicate) for each construct (empty pJL-TRBO vector (EV), pRC-eGFP (pRC), pJL-TRBO-eGFP (pJL-TRBO) and pR5-eGFP (pR5). Lane M shows a BioRad PrecisionPlus dual colour marker. The RbcL can be seen at 50 kDa (red boxes show quantified regions) and the eGFP can be seen at 27 kDa (blue boxes show quantified regions). **B.** Uncropped Wastern blot image (anti-GFP; 10µg protein loaded per lane) showing total protein expression (in triplicate) for each construct (empty pJL-TRBO vector (EV), pRC-eGFP (pRC), pJL-TRBO-eGFP (pJL-TRBO) and pR5-eGFP (pR5). Lane M shows a BioRad PrecisionPlus dual colour marker. The eGFP can be seen at 27 kDa (green boxes show quantified regions). **C.** Histogram showing quantification of eGFP:RbcL signal from Panel A (with band background for EV lanes subtracted). pRC-eGFP and pR5-eGFP show a significantly higher ratio than pJL-TRBO (p < 0.01). The difference in expression between pRC-eGFP and pR5-eGFP was not statistically significant. Individual data points are shown. D – Box plot showing Odyssey Clx quantification of the 27 kDa eGFP bands. A one way ANOVA showed a statistically significant difference between means (F_(3, 8)_ = 143.2, p = 2.73x10^-7^). A Tukey test shows that pRC-eGFP, pR5-eGFP and pJL-TRBO-eGFP have significantly higher fluorescence than the empty vector control (p < 0.0001). Both pRC-eGFP and pR5-eGFP show significantly higher fluorescence than the parent pJL-TRBO-eGFP vector (p < 0.001). The difference in expression between pRC-eGFP and pR5-eGFP was not statistically significant (p = 0.9998). Each box plot shows the interquartile range for each dataset and the error bars show the standard error of the mean. Individual data points are shown as black dots and the centreline in each plot shows the mean. Significance values are: ns = not significant, * = p < 0.05, ** = p < 0.01, *** = p < 0.001, **** = p < 0.0001.

**C**


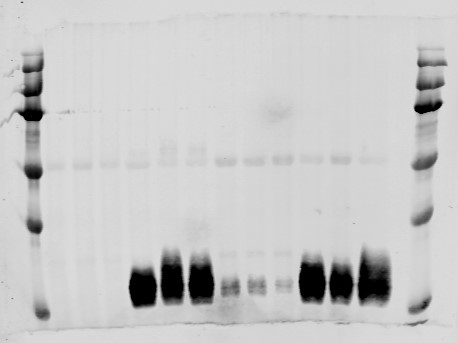


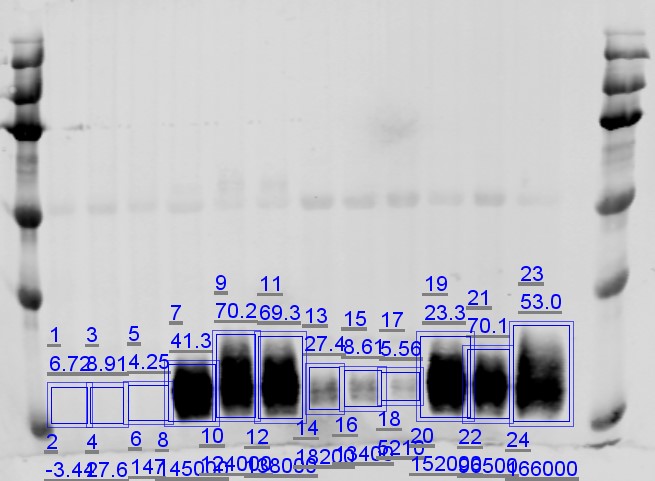


**Figure S4. Original Western Blot images (uncropped) used for Figure 5**. Upper panel shows original image. Lower panel shows regions used for band quantification.

**DNA sequences**

**CaMV 35S Promoter**

GAATTCCAATCCCACAAAAATCTGAGCTTAACAGCACAGTTGCTCCTCTCAGAGCAGAATCGGGTATTCAACACCCTCATATCAACTACTACGTTGTGTATAACGGTCCACATGCCGGTATATACGATGACTGGGGTTGTACAAAGGCGGCAACAAACGGCGTTCCCGGAGTTGCACACAAGAAATTTGCCACTATTACAGAGGCAAGAGCAGCAGCTGACGCGTACACAACAAGTCAGCAAACAGACAGGTTGAACTTCATCCCCAAAGGAGAAGCTCAACTCAAGCCCAAGAGCTTTGCTAAGGCCCTAACAAGCCCACCAAAGCAAAAAGCCCACTGGCTCACGCTAGGAACCAAAAGGCCCAGCAGTGATCCAGCCCCAAAAGAGATCTCCTTTGCCCCGGAGATTACAATGGACGATTTCCTCTATCTTTACGATCTAGGAAGGAAGTTCGAAGGTGAAGGTGACGACACTATGTTCACCACTGATAATGAGAAGGTTAGCCTCTTCAATTTCAGAAAGAATGCTGACCCACAGATGGTTAGAGAGGCCTACGCAGCAAGTCTCATCAAGACGATCTACCCGAGTAACAATCTCCAGGAGATCAAATACCTTCCCAAGAAGGTTAAAGATGCAGTCAAAAGATTCAGGACTAATTGCATCAAGAACACAGAGAAAGACATATTTCTCAAGATCAGAAGTACTATTCCAGTATGGACGATTCAAGGCTTGCTTCATAAACCAAGGCAAGTAATAGAGATTGGAGTCTCTAAAAAGGTAGTTCCTACTGAATCTAAGGCCATGCATGGAGTCTAAGATTCAAATCGAGGATCTAACAGAACTCGCCGTCAAGACTGGCGAACAGTTCATACAGAGTCTTTTACGACTCAATGACAAGAAGAAAATCTTCGTCAACATGGTGGAGCACGACACTCTGGTCTACTCCAAAAATGTCAAAGATACAGTCTCAGAAGATCAAAGGGCTATTGAGACTTTTCAACAAAGGATAATTTCGGGAAACCTCCTCGGATTCCATTGCCCAGCTATCTGTCACTTCATCGAAAGGACAGTAGAAAAGGAAGGTGGCTCCTACAAATGCCATCATTGCGATAAAGGAAAGGCTATCATTCAAGATCTCTCTGCCGACAGTGGTCCCAAAGATGGACCCCCACCCACGAGGAGCATCGTGGAAAAAGAAGAGGTTCCAACCACGTCTACAAAGCAAGTGGATTGATGTGACATCTCCACTGACGTAAGGGATGACGCACAATCCCACTATCCTTCGCAAGACCCTTCCTCTATATAA

**Synthetic 5’ UTR**

TTTAAGAGACGCAACCACAACGCTCTAACGCAATCAATCTACATTATATTAAACGTCTCTAAAA

**CPMV 3’ UTR**

TTAACTCTGGTTTCATTAAATTTTCTTTAGTTTGAATTTACTGTTATTCGGTGTGCATTTCTATGTTTGGTGAGCGGTTTTCTGTGCTCAGAGTGTGTTTATTTTATGTAATTTAATTTCTTTGTGAGCTCCTGTTTAGCAGGTCGTCCCTTCAGCAAGGACACAAAAAGATTTTAATTTTATT

**EU-NbAct double Terminator**

GCTTAAAGCAGAATGCTGAGCTAAAAGAAAGGCTTTTTCCATTTTCGAGAGACAATGAGAAAAGAAGAAGAAGAAGAAGAAGAAGAAGAAGAAGAAAAGAGTAAATAATAAAGCCCCACAGGAGGCGAAGTTCTTGTAGCTCCATGTTATCTAAGTTATTGATATTGTTTGCCCTATATTTTATTTCTGTCATTGTGTATGTTTTGTTCAGTTTCGATCTCCTTGCAAAATGCAGAGATTATGAGATGAATAAACTAAGTTATATTATTATACGTGTTAATATTCTCCTCCTCTCTCTAGCTAGCCTTTTGTTTTCTCTTTTTCTTATTTGATTTTCTTTAAATCAATCCATTTTAGGAGAGGGCCAGGGAGTGATCCAGCAAAACATGAAGATTAGAAGAAACTTCCCTCTTTTTTTTCCTGAAAACAATTTAACGTCGAGATTTATCTCTTTTTGTAATGGAATCATTTCTACAGTTATGACATACAGCATTCCCAGAAAGAGAAACAGAAGAAATATACAAACTTTCATTTTGAGAGCAGCACCTCGTCTATTGATTGCAGATAATATGCTTCTCATTTGTATTTCCTTTTGATTATTTTTGTTTCTATCCCTTTGTTTGAGTCAATCTCAAATATTCGGTCATTGTTGGTATGAAAAATCAAGCAGTTCATGTTAAGAGTCAATTTAAAATTAATATTTTTATATAGAGTTGTATGTGAAATGATGTTGTGATTTGGTATATATGGATAAAGAGCTTGTCAGTTCATTTTGGTCTCATTTTTTTGGTATCCAAATAAGAAACACAAAAGGGATATGTCCCTCTACTATCAAATATTAGTTATAAGTATTCATGTTATACTATTCGATATTTTCTACCCCAATCGTTACCTATTTAAAAGTATTTACCCCTCCATCTATCAAACCCCTGGACCCAGCTTTCCTATTACATGTGGCTTCATCTTAAGCCCCCAAACCTTTTTCTTATTTTTGATTTTTAAAGGCTCATCTTAAAATTTATTACTCAAATTAATACCTCTTAATAACCCACCTCAAGGACCCAGTAATTAAATATCCAATTAGCTCCAGTAATTGGGGTTCATATTAGCTCCAGTCTTAAATTTTAAAGGCGATGATCGTATTCCTCCACTTGGTTCATTTATACTCAAAGAATACTCAATGTCTTTAGTGTTTAGATAACTTTTTGTAAATCATATAGATTGTTTTAACAAAAAACAATTCAATAGTAGATTTTCACATGAAAGTTACATAAAAATTCTTTAAAATTACTTTCTCAAAAAATTGTTCCAAACATATTATCCCACAATTAAACTCAATCTGTTTTTCGAAACCTAAATCAAAACCAATCCAACTACCTTATATAATATATAATCAATACATTGTAAAGAACTGCATGTTCTTTTAAATTTTGGGGGCAAAGTTATTCCGTACGTTCACACATGTACTAATAGGAGGTAATAAATGATATGTGAAACAATCGAGGTGTAAACAAGCTAGCATGGTA

**RB7 MAR**

GGTATCGATTAAAAATCCCAATTATATTTGGTCTAATTTAGTTTGGTATTGAGTAAAACAAATTCGAACCAAACCAAAATATAAATATATAGTTTTTATATATATGCCTTTAAGACTTTTTATAGAATTTTCTTTAAAAAATATCTAGAAATATTTGCGACTCTTCTGGCATGTAATATTTCGTTAAATATGAAGTGCTCCATTTTTATTAACTTTAAATAATTGGTTGTACGATCACTTTCTTATCAAGTGTTACTAAAATGCGTCAATCTCTTTGTTCTTCCATATTCATATGTCAAAATCTATCAAAATTCTTATATATCTTTTTCGAATTTGAAGTGAAATTTCGATAATTTAAAATTAAATAGAACATATCATTATTTAGGTATCATATTGATTTTTATACTTAATTACTAAATTTGGTTAACTTTGAAAGTGTACATCAACGAAAAATTAGTCAAACGACTAAAATAAATAAATATCATGTGTTATTAAGAAAATTCTCCTATAAGAATATTTTAATAGATCATATGTTTGTAAAAAAAATTAATTTTTACTAACACATATATTTACTTATCAAAAATTTGACAAAGTAAGATTAAAATAATATTCATCTAACAAAAAAAAAACCAGAAAATGCTGAAAACCCGGCAAAACCGAACCAATCCAAACCGATATAGTTGGTTTGGTTTGATTTTGATATAAACCGAACCAACTCGGTCCATTTGCACCCCTAATCATAATAGCTTTAATATTTCAAGATATTATTAAGTTAACGTTGTCAATATCCTGGAAATTTTGCAAAATGAATCAAGCCTATATGGCTGTAATATGAATTTAAAAGCAGCTCGATGTGGTGGTAATATGTAATTTACTTGATTCTAAAAAAATATCCCAAGTATTAATAATTTCTGCTAGGAAGAAGGTTAGCTACGATTTACAGCAAAGCCAGAATACAAAGAACCATAAAGTGATTGAAGCTCGAAATATACGAAGGAACAAATATTTTTAAAAAAATACGCAATGACTTGGAACAAAAGAAAGTGATATATTTTTTGTTCTTAAACAAGCATCCCCTCTAAAGAATGGCAGTTTTCCTTTGCATGTAACTATTATGCTCCCTTCGTTACAAAAATTTTGGACTACTATTGGGAACTTCTTCTGAAAATAGTGCCTAGGCGCT

**eGFP coding sequence**

ATGAGCAAGGGCGAGGAGCTGTTCACCGGGGTGGTGCCCATCCTGGTCGAGCTGGACGGCGACGTAAACGGCCACAAGTTCAGCGTGTCCGGCGAGGGCGAGGGCGATGCCACCTACGGCAAGCTGACCCTGAAGTTCATCTGCACCACCGGCAAGCTGCCCGTGCCCTGGCCCACCCTCGTGACCACCCTGACCTACGGCGTGCAGTGCTTCAGCCGCTACCCCGACCACATGAAGCAGCACGACTTCTTCAAGTCCGCCATGCCCGAAGGCTACGTCCAGGAGCGCACCATCTTCTTCAAGGACGACGGCAACTACAAGACCCGCGCCGAGGTGAAGTTCGAGGGCGACACCCTGGTGAACCGCATCGAGCTGAAGGGCATCGACTTCAAGGAGGACGGCAACATCCTGGGGCACAAGCTGGAGTACAACTACAACAGCCACAACGTCTATATCATGGCCGACAAGCAGAAGAACGGCATCAAGGTGAACTTCAAGATCCGCCACAACATCGAGGACGGCAGCGTGCAGCTCGCCGACCACTACCAGCAGAACACCCCCATCGGCGACGGCCCCGTGCTGCTGCCCGACAACCACTACCTGAGCACCCAGTCCAAGCTGAGCAAAGACCCCAACGAGAAGCGCGATCACATGGTCCTGCTGGAGTTCGTGACCGCCGCCGGGATCACTCTCGGCATGGACGAGCTGTACAAGTCCGGACTCAGATCTCACCATCATCATCACCACCATCACTGA
